# P-1338. Don't You Forget About Me: Older Patients with Substance Use Disorder -- Have We Left Them Behind on Screening?

**DOI:** 10.1093/ofid/ofae631.1515

**Published:** 2025-01-29

**Authors:** Diana Yu, Amber C Streifel, Cara D Varley

**Affiliations:** Doernbecher Children's Hospital/OHSU Healthcare, Portland, Oregon; Oregon Health and Science University, Portland, Oregon; Oregon Health & Science University, Portland, OR

## Abstract

**Background:**

Oregon has seen increasing incidence of sexually transmitted infections [e.g. HIV, syphilis, gonorrhea, chlamydia (GC/CHL), hepatitis C (HCV)], as well as outbreaks associated with vaccine preventable infections [e.g. hepatitis A (HAV), B (HBV)]. Limited data are available on routine screening and vaccination rates despite guidelines expanding screening, especially in high-risk patients such as those with substance use disorder (SUD). This study aims to determine rates of screening in persons with SUD.

**Table 1.**
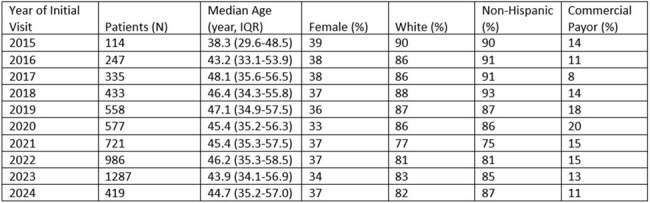
Patient Demographics by Year of First Consultation

**Methods:**

Single-center, retrospective cohort study in Portland, Oregon, USA. Patients aged 12-100 years with an Addiction Medicine consultation between January 1, 2015-March 31, 2024 were included. Data abstracted included patient demographics, payor status, and laboratory results for GC/CHL, syphilis, HAV, HBV, and HCV, and HIV. Chi-squared test was used to analyze differences in testing rate testing by age group.

**Table 2.**
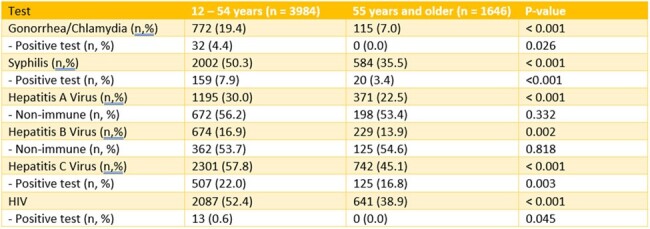
Screening Rates and Results by Age Group

**Results:**

A total of 5630 patients met inclusion criteria (Table 1). For GC/CHL screening, 887 patients had testing; 32 and 24 of 1228 tests were positive, respectively. For syphilis screening, 2364 patients were tested; 260 of 3539 tests were reactive. HAV serology was performed in 1566 patients; 694 were immune, 596 non-immune, 2 with active infection, and 274 were IgM negative at initial testing. There were 3998 tests for HBV; however, 2709 were incomplete panels (e.g. missing HBVsAb). Of the 903 patients with a full panel, 264 were immune, 92 had prior infection, 11 with acute/chronic infection, and 536 non-immune at initial testing. 4206 HCV tests were performed in 3043 patients with 632 new or recent diagnoses during the study period. 4603 HIV screening tests were performed in 2728 patients, with 13 new diagnoses. Patients aged 55 years and older were less likely to receive testing (Table 2).

**Conclusion:**

Despite guidelines recommending frequent screening in high-risk patients, it appears to be limited at this institution. Furthermore, screening tests revealed several opportunities for healthcare intervention (e.g. vaccination) thus efforts should be made to secure access to testing. Future directions should include ordersets to ensure proper testing and follow through of results, as well as education and feedback to providers of screening results.

**Disclosures:**

**All Authors**: No reported disclosures

